# L-shaped association between the GA/HbA1c ratio and all-cause mortality in U.S. adults with NAFLD: a cross-sectional study from the NHANES 1999–2004

**DOI:** 10.1186/s12902-024-01568-7

**Published:** 2024-03-11

**Authors:** Zhaofu Zhang, Hao Wang, Mingyu Chen, Youpeng Chen

**Affiliations:** Department of Infectious Diseases, The Seventh Affiliated Hospital, Sun Yat-sen University, Shenzhen, 518107 China

**Keywords:** Nonalcoholic fatty liver disease, Glycated albumin to HbA1c ratio, National Health and Nutrition Examination Survey, Mortality

## Abstract

**Objective:**

It is currently unclear whether there is a relationship between the ratio of glycated albumin to hemoglobin A1c (GA/HbA1c) and mortality in individuals diagnosed with nonalcoholic fatty liver disease (NAFLD). The primary objective of the study was to investigate the relationship between the GA/HbA1c ratio and all-cause mortality in adults with NAFLD in the U.S.

**Methods:**

The investigation included a total of 5,295 individuals aged ≥ 18 years who were diagnosed with NAFLD, these individuals were selected from the National Health and Nutrition Examination Survey conducted between 1999 and 2004. To evaluate the outcomes of death, the researchers relied on National Death Index (NDI) records up to December 31, 2019. To better understand the nonlinear relationship between the GA/HbA1c ratio and mortality among individuals with NAFLD, this study employed both subgroup and sensitivity analyses. Furthermore, Cox proportional hazards models and two-part Cox proportional hazards model were utilized.

**Results:**

The study included a total of 5,295 adult patients with NAFLD in the U.S. During a median follow-up period of 16.9 years, there were 1,471 recorded deaths, including 419 cardiovascular deaths. After accounting for various factors, a higher GA/HbA1c ratio exhibited a positive and nonlinear association with an increased risk of all-cause mortality in patients with NAFLD. Furthermore, the study revealed an L-shaped relationship between the GA/HbA1c ratio and all-cause mortality, with the inflection point occurring at a GA/HbA1c ratio of 2.21. When the GA/HbA1c ratio exceeded 2.21, each 1-unit increase in the ratio was associated with a 33% increase in the adjusted hazard ratio (HR 1.33; 95% CI 1.14, 1.60) for all-cause mortality.

**Conclusions:**

A nonlinear correlation between the ratio of GA to HbA1c and all-cause mortality was observed in U.S. adults with NAFLD. In addition, an elevated GA/HbA1c ratio was linked to an increased risk of all-cause mortality in these patients.

## Introduction

The most prevalent chronic liver disease in developed nations is nonalcoholic fatty liver disease (NAFLD), which has a prevalence of approximately 13–28% worldwide [1]. A meta-analysis, which included 5,399,254 people, showed a global combined prevalence of NAFLD of 29.8% in 2019 [2]. According to United States (US) research, the incidence of NAFLD rose from 29.5% in 1999–2000 to 40.3% in 2015–2016 [3]. NAFLD is said to be responsible for approximately 8% of fatalities in the U.S. from all causes and for more than 30% of deaths specifically related to diabetes and liver disease [4]. NAFLD also has the potential to lead to further systemic disorders, such as diabetes, insulin resistance, and heart disease, in addition to liver impairment [5]. Due to the increasing incidence of NAFLD, it is imperative to identify reliable predictors of mortality resulting from NAFLD among the general population.

Glycated hemoglobin (HbA1c) is a typical glycated protein widely used for evaluating blood glucose control [6]. It is formed through the interaction between hemoglobin and blood glucose and can reflect the average blood glucose levels over a period of 2–3 months [7]. Previous study indicated that HbAlc has a positive correlation with NAFLD in non diabetes patients [8, 9]. However, HbA1c is influenced by red blood cell lifespan. Glycated albumin (GA) serves as an intermediate marker of glycemic control in diabetic patients and complements the measurement of HbA1c. Since, GA levels are not affected by red blood cell lifespan, when there are issues with interpreting HbA1c (e.g., hemoglobin variants, iron deficiency, or anemia), GA levels can serve as an effective alternative to HbA1c. The ratio of glycated albumin to glycated hemoglobin (GA/HbA1c), which may be used to monitor blood glucose changes in diabetic patients independent of their type of diabetes or level of glycemic regulation, has gained widespread acceptance in recent years [10, 11]. Diabetes-related problems such retinopathy, atherosclerosis, and nephropathy have been linked to high GA/HbA1c ratios [12–14]. There are several previous studies about the GA/HbA1c ratio and NAFLD incidence. For instance, research has demonstrated that individuals with type 2 diabetes mellitus (T2DM) combined with NAFLD exhibit considerably lower GA/HbA1C ratios than do those without NAFLD [15]. A number of studies have shown that a higher GA/HbA1C ratio is strongly associated with the degree of liver fibrosis in individuals, regardless of the presence of chronic hepatitis [16–18]. However, it is unclear whether there is a correlation between the GA/HbA1c ratio and mortality in patients suffering from NAFLD.

Our primary objective was to investigate the relationship between the GA/HbA1c ratio and all-cause mortality in a nationally representative sample of U.S. NAFLD adults from the National Health and Nutrition Examination Survey (NHANES 1999–2004) in response to these knowledge gaps.

## Methods

### Study design and participants

The NHANES is a continuous, nationwide, cross-sectional series of surveys that measures the nutritional and physical health of the non-institutionalized U.S. civilian population using a multistage, sophisticated sampling approach [19]. No additional ethics approval is required for the NHANES, as all the surveys and studies were conducted under the supervision and direction of the Centers for Disease Control and Prevention. GA information was only available from the NHANES for the 1999 to 2004 survey cycle, as only participants from this cycle were selected for this study. All of the subjects were followed up on a prospective basis until 31 December 2019. Among the 31,126 participants in the three cycles, we excluded patients who were under the age of 18 years, lacked complete laboratory data such as GA and HbAlc, and lacked information about hepatic steatosis index (HSI) and liver steatosis from other causes, such as viruses and alcohol use disorder.

**Fig. 1:**
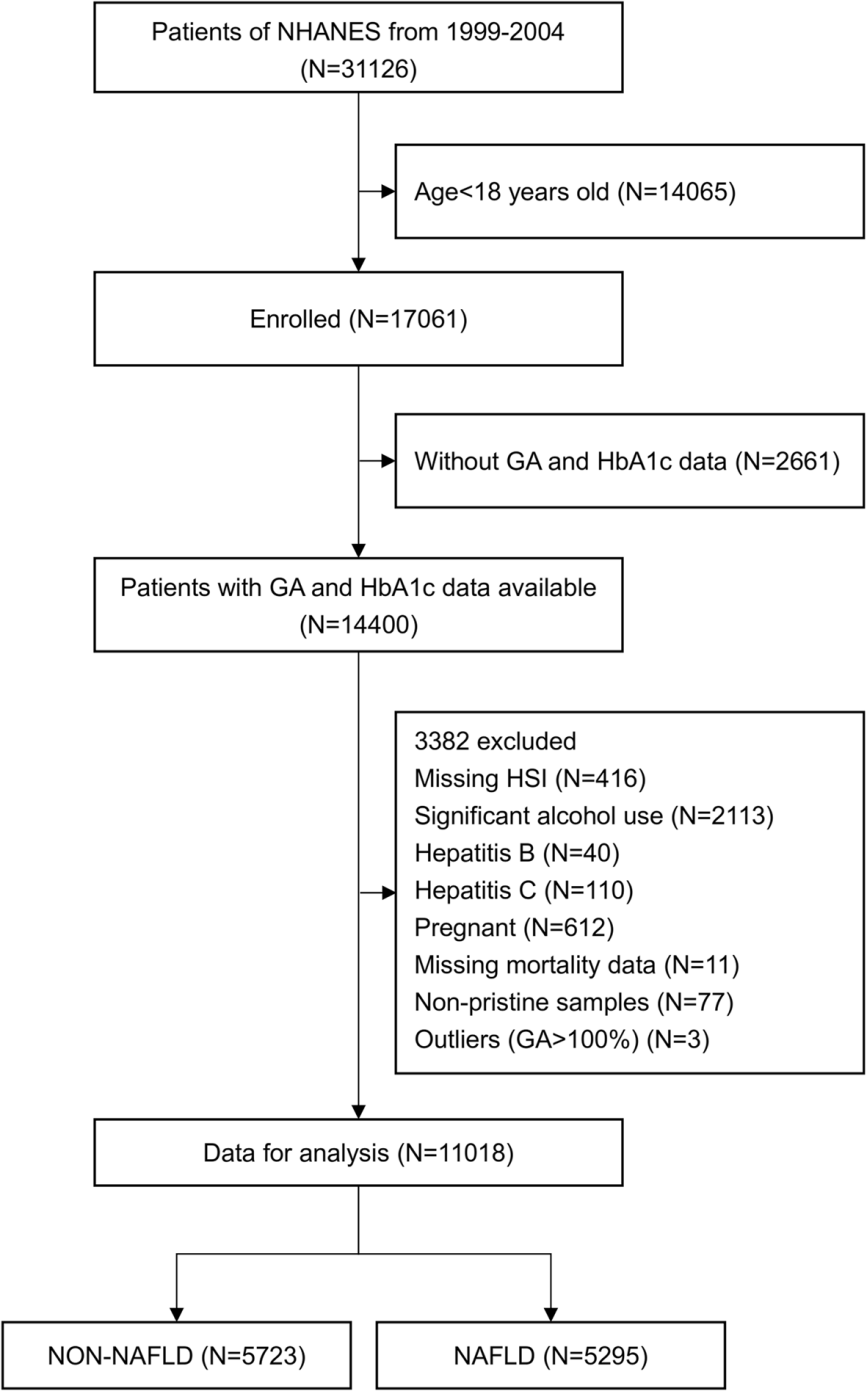
Flow-chart of the enrolled participants. NHANES, National Health and Nutrition Examination Survey; GA, Glycated albumin; HbA1c, hemoglobin A1c; HSI, Hepatic steatosis index

The inclusion criteria were as follows: (1) ≥ 18 years of age, (2) complete anthropometric and laboratory data, and (3) available HSI calculations. The exclusion criteria included (1) pregnancy; (2) other causes of liver disease, such as significant alcohol use (males with > 140 g/week or females > 70 g/week), carrying or infected by viral hepatitis; (3) missing mortality data or non-pristine samples; and (4) (non-pristine samples) that resulted in incredible GA outliers (GA > 100%). Finally, 5,295 adult participants with an HSI > 36 were included in the analysis. The flowchart of this study is shown in Fig. 1.

### Definition of the GA/HbA1 ratio

In 2018–2020, tests of GA were conducted on excess sera specimens from NHANES 1999–2004 held at the University of Maryland School of Medicine in Baltimore, Maryland. GA was measured using a complex method by Asahi Kasei Pharma (Lucica-GA-L) adapted to the Siemens Dimension Vista 1500 (Siemens Healthcare Diagnostics). The following equation was used to calculate the ratio of GA to total albumin: [(GA concentration in g/dL/serum albumin concentration in g/dL) 100/1.14] + 2.9 [20]. HbAlc was tested by the Primus CLC330 GHb Analyzer (Primus, Kansas City, MO), which is an HPLC procedure that uses a short boronate affinity resin column to quickly separate glycated and nonglycated hemoglobins.

### Definition of NAFLD

The HSI was used to identify NAFLD using the following formula: HSI = 8 × (alanine aminotransferase (ALT)/aspartate aminotransferase (AST) ratio) + body mass index (+ 2 for female; +2 for diabetes) [21]. Values < 30 were used to rule-out steatosis while values > 36 indicated steatosis [22].

### Definition of NAFLD-related advanced fibrosis

The NAFLD Fibrosis Score (NFS) was used to categorize NAFLD patients with advanced fibrosis as high risk (NFS > 0.676), intermediate risk (NFS 0.676 to -1.455) or low risk (NFS < -1.455) and was calculated using the following formula: NFS = -1.675 + 0.037 × age (years) + 0.094 × body mass index (BMI) (kg/m^2^) + 1.13 × impaired fasting plasma glucose (FPG)/diabetes mellitus (DM) (yes = 1, no = 0) + 0.99 × AST/ALT ratio − 0.013 × platelet count (×109/L) − 0.66× albumin (g/dl) [23].

### Assessment of covariates

According to the use of the NHANES, we divided the participants into 5 races and ethnicities. In addition, educational levels were divided into three categories: less than high school, high school, or more than high school. Marital status was divided into married and unmarried; household income was classified as low (poverty income ratio (PIR) < 1), medium (1 ≤ PIR ≤ 3), high (PIR > 3), or not recorded; and smoking status was categorized as never smoked, ever smoked and currently smoked. Physical activity was categorized as none, moderate or vigorous [24]. Self-reported history, antihypertensive drug use, or blood pressure ≥ 140/90 mmHg were used to diagnose hypertension [25]. The diagnosis of diabetes was based on the patient’s past history of the disease, treatment with oral hypoglycemic agents or insulin, and a fasting blood glucose level ≥ 126 mg/dL or a glycated hemoglobin A1c level ≥ 6.5% [26]. Obesity was defined as a BMI ≥ 30 kg/m^2^. A self-reported history of any malignancy was used to define cancer [27]. The Chronic Kidney Disease Epidemiology Collaboration (CKD-EPI) algorithm was used to determine the estimated glomerular filtration rate (eGFR) [28]. Laboratory biochemical tests in this study included GA(%), HbAlc(%), albumin (mg/dl), ALT (IU/L), AST (IU/L), serum creatinine (mg/dl), fasting blood glucose (mg/dl). Except for fasting blood glucose, which is performed in the fasting state, other biochemical tests are performed in the non-fasting state. Specific descriptions of biochemical tests are available on the official CDC website (www.cdc.gov/nchs/nhanes).

### Assessment of mortality

We linked baseline NHANES data from 1999 to 2004 with fatality data reported in the National Death Index as of December 31, 2019, to assess the mortality status. The results of our study included all-cause mortality and cardiovascular disease-specific mortality (codes I00-I09 and I11, I13, I20-I51 and I60-I69).

### Statistical analysis

We took into account weights in all subsequent analyses to obtain national estimates because the NHANES utilizes a statistically complex sampling technique to select a representative sample of the U.S. population. Descriptive statistics for continuous variables are expressed as weighted means (standard errors [SEs]). Descriptive statistics for categorical variables are expressed as weighted percentages (SEs).

In this study, we used Cox proportional hazards model to evaluate the associations between the GA/HbA1c ratio and cardiovascular and all-cause mortality among individuals with NAFLD while accounting for possible confounders. Model 1 was unadjusted for any covariates. Model 2 was adjusted for age, sex, and race. Model 3 included adjustments for every factor in Model 2, as did marital status, education, the PIR, BMI, eGFR, NFS, smoking status, physical activity, diabetes status, hypertension status, cardiovascular disease (CVD), and cancer.

Age groups (< 60 years or ≥ 60 years), sex (male or female), type 2 diabetes status (yes or no), obesity status (BMI ≥ 30 kg/m^2^ or not), hypertension status (yes or no), and NFS were used to perform stratification analyses. To ensure result stability, we conducted two sensitivity analyses. First, to prevent the impact of reverse causation on the results, those who died within 2 years of follow-up were excluded. Second, less than 10% of the overall PIR data were missing, and to exclude the effect of missing data on the results, these data were excluded and reanalyzed. Stata 16.0 statistical analysis software (StataCorp, College Station, TX, USA) and R 4.2.1 software (http://www.R-project.org, The R Foundation, Austria) were used for all analyses, considering the complex survey design and weights of the NHANES. A two-tailed *P* value < 0.05 indicated statistical significance.

## Results

### Features of the study population

A total of 5,295 individuals with NAFLD were included; 46.8% were male, and the average (SE) age was 48.1 (0.2) years. During a median follow-up period of 16.9 years, 1,471 deaths occurred, 419 of which were cardiovascular deaths. A higher GA/HbA1c ratio was linked to a greater likelihood diabetes and cancer, as well as a lower likelihood of being female and non-Hispanic White (*P* < 0.05). Furthermore, these participants were older, and had higher levels of NFS and lower BMIs and eGFRs (*P* < 0.05). Table 1 displays the basic features of the study population based on the GA/HbA1c ratio.

**Table 1:** Baseline characteristics of the population, according to tertiles of GA/HbA1c ratio

Variables	Overall	Tertiles of GA/HbA1c ratio			* P* value
		T1	T2	T3	
Age, years	48.1 (0.2)	46.7 (0.4)	47.8 (0.4)	49.8 (0.5)	< 0.001
Male, %	46.8 (0.9)	49.1 (1.5)	47.2 (1.5)	43.8 (1.5)	0.006
Race/ethnicity, %					< 0.001
Mexican American	6.7 (0.03)	7.3 (0.5)	7.3 (0.5)	5.4 (0.4)	
Other Hispanic	5.8 (0.4)	5.6 (0.7)	5.8 (0.7)	5.9 (0.8)	
Non-Hispanic White	72.5 (0.7)	75.9 (1.1)	72.1 (1.2)	69.3 (1.3)	
Non-Hispanic Black	10.9 (0.4)	8.3 (0.6)	10.4 (0.6)	14.1 (0.8)	
Other race	4.1 (0.4)	2.9 (0.5)	4.4 (0.7)	5.2 (0.8)	
Married, %	64.0 (0.8)	65.2 (1.4)	62.5 (1.4)	64.4 (1.5)	0.223
Educational level, %					< 0.001
< high school	20.8 (0.6)	20.3 (1.5)	19.5 (1.5)	22.7 (1.5)	
High school	27.5 (0.8)	31.1 (1.5)	25.3 (1.5)	26.0 (1.5)	
> high school	51.7 (0.9)	48.6 (1.1)	55.2 (1.2)	51.3 (1.3)	
Diabetes mellitus, %	16.0 (0.6)	14.3 (1.0)	12.8 (0.9)	21.1 (1.2)	< 0.001
Hypertension, %	45.0 (0.9)	46.9 (1.5)	44.2 (1.5)	43.7 (1.5)	0.115
CVD, %	10.6 (0.5)	10.3 (0.9)	9.8 (0.8)	11.9 (0.9)	0.119
Cancer, %	8.0 (0.4)	6.9 (0.7)	7.6 (0.7)	9.6 (0.9)	0.009
Smoker, %					< 0.001
Never	55.4 (0.9)	50 (1.5)	57.3 (1.5)	59.4 (1.5)	
Former	28.0 (0.8)	28.3 (1.3)	26.8 (1.3)	28.8 (1.4)	
Current	16.6 (0.7)	21.7 (1.3)	15.9 (1.1)	11.8 (1.0)	
Physical activity,%					< 0.001
Never	39.1 (0.8)	39.0 (1.4)	37.7 (1.4)	40.6 (1.5)	
Moderate	31.0 (0.8)	33.9 (1.4)	29.3 (1.4)	29.7 (1.4)	
Vigorous	29.9 (0.8)	27.2 (1.4)	33.0 (1.5)	29.7 (1.4)	
Poverty-income ratio,%					0.372
Low	11.4 (0.5)	11.8 (0.9)	10.8 (0.8)	11.6 (0.9)	
Moderate	34.5 (0.8)	36.0 (1.4)	33.0 (1.4)	34.2 (1.4)	
High	47.3 (0.9)	45.6 (1.5)	49.3 (1.5)	47.1 (1.6)	
Not recorded	6.8 (0.4)	6.5 (0.7)	6.9 (0.8)	7.1 (0.7)	
Advanced fibrosis risk stratification, %					< 0.001
NFS < -1.455 (%)	67.8 (0.8)	69.0 (1.3)	70.6 (1.3)	63.6 (1.4)	
NFS -1.455 to 0.676 (%)	27.8 (0.7)	27.4 (1.3)	26.4 (1.3)	29.6 (1.3)	
NFS > 0.676 (%)	4.4 (0.3)	3.6 (0.5)	3.0 (0.4)	6.8 (0.6)	
BMI, kg/m^2^	32.37 (0.10)	33.88 (0.19)	32.23 (0.16)	30.96 (0.14)	< 0.001
eGFR, mL/min/1.73 m^2^	97.37 (0.38)	98.85 (0.60)	97.56 (0.64)	95.39 (0.71)	< 0.001

Data were presented as weighted mean (SE) for continuous variables and weighted percentages (SE) for categorical variables

*Abbreviation: CVD* Cardiovascular disease, *NFS *Nafld fibrosis score, *BMI *Body mass index, *eGFR *Estimated glomerular filtration rate, *HbA1c *Hemoglobin A1c, *GA *Glycated albumin

### GA/HbA1c ratio and mortality

**Fig. 2:**
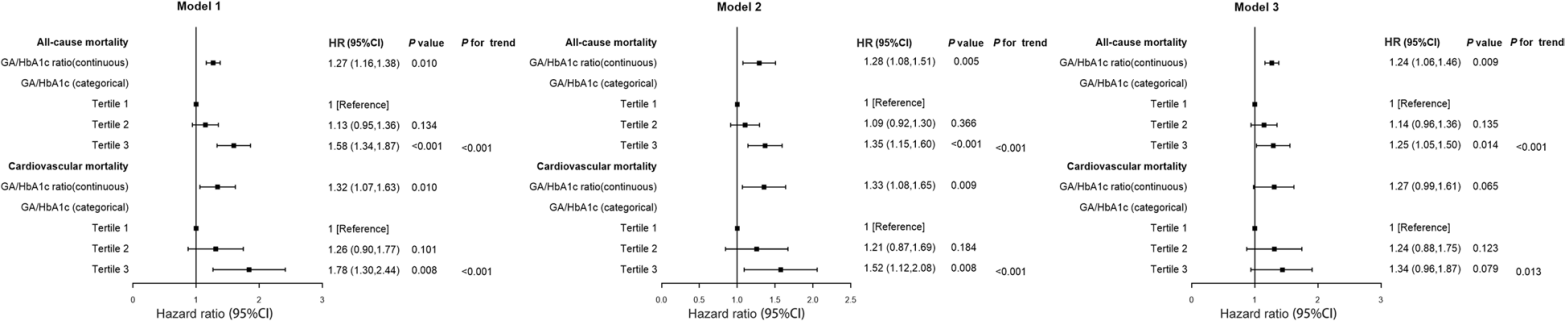
HRs (95% CIs) for mortality according to GA/HbA1c ratio among participants with NAFLD. Model 1: unadjusted. Model 2: adjusted for age, sex and race. Model 3: adjusted for Model 2 plus education level, marital status, poverty income ratio, hypertension, diabetes, cardiovascular disease, cancer, smoker, physical activity, estimated glomerular filtration rate, body mass index and nafld fibrosis score

According to the tertiles of the GA/HbA1c ratio, NAFLD patients were divided into three groups: T1 ≤ 2.29, 2.29 < T2 ≤ 2.53, and T3 > 2.53. There were 1,682, 1,822, and 1,791 individuals in each group, respectively. Variables affecting clinical prognosis were selected for inclusion in Cox multivariate regression models based on previous experience. Model 1 was unadjusted; Model 2 was adjusted for age, sex and race; and Model 3 was adjusted for Model 2 plus education level, marital status, PIR, hypertension, diabetes, CVD, cancer, smoking status, physical activity, eGFR, BMI and NFS. As shown in Fig. 2, we found that all-cause mortality was significantly greater in the NAFLD group in the third tertile of the GA/HbA1c ratio than in the first tertile (HR 1.58, 95% CI 1.34–1.87; *P* < 0.001). After adjusting for confounding factors, this relationship still existed (HR 1.25, 95% CI 1.05–1.50; *P* = 0.014). However, there was no such connection between the GA/HbA1c ratio and cardiovascular mortality (HR 1.34, 95% CI 0.96–1.87; *P* = 0.079). Furthermore, after performing continuous analyses, it was found that an increased GA/HbA1c ratio was significantly associated with a higher mortality rate from all causes in Model 3 (HR 1.24, 95% CI 1.06–1.46; *P* = 0.009). However, the relationship with cardiovascular mortality was still not significant in Model 3 (HR 1.27, 95% CI 0.99–1.61, *P* = 0.065).

### Detection of nonlinear relationships

**Fig. 3:**
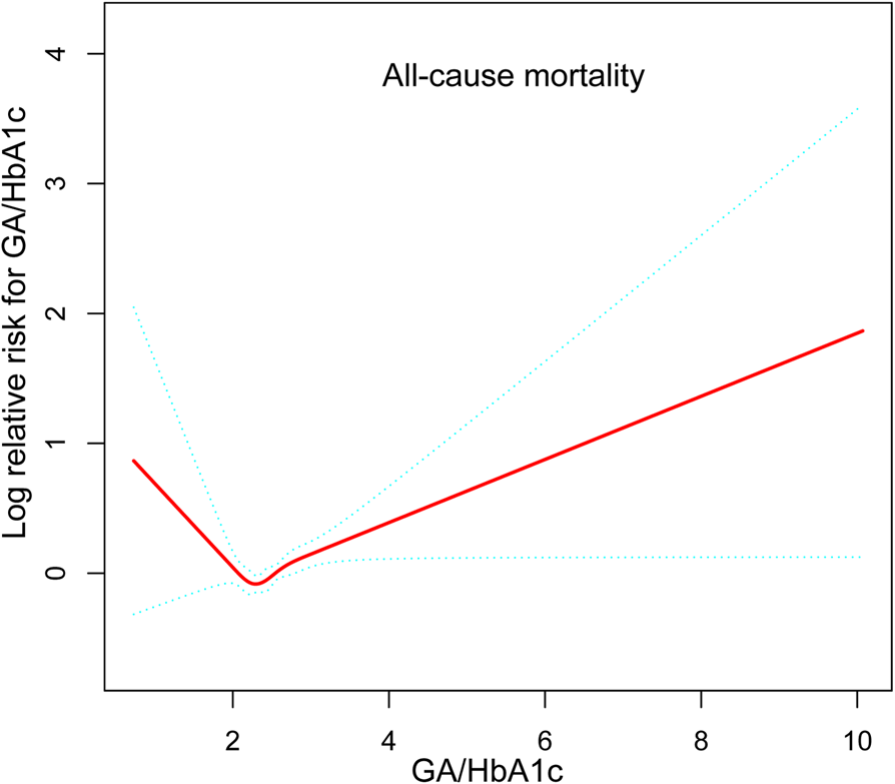
Association between GA/HbAlc ratio and all-cause mortality of individuals with NAFLD. Adjusted for age, sex, race, education level, marital status, poverty income ratio, hypertension, diabetes, cardiovascular disease, cancer, smoker, physical activity, estimated glomerular filtration rate, body mass index and nafld fibrosis score. The solid and dotted lines represent the estimated values and their corresponding 95% CIs, respectively

We also performed a nonlinear test as described in a previous study [29]. Using a generalized additive model and smooth curve fitting (penalized spline approach), we identified an L-shaped connection between the GA/HbA1c ratio and all-cause mortality (Fig. 3). Then, we further searched for nonlinear relationship and inflection point between the GA/HbA1c ratio and all-cause mortality through the Cox proportional hazards model combined with a two-way Cox proportional hazards model. The inflection point at which all-cause mortality occurred in participants with NAFLD was a GA/HbA1c ratio of 2.21. When the GA/HbA1c ratio was greater than 2.21, for every 1-unit increase in the GA/HbA1c ratio, the corresponding adjusted HR for all-cause mortality increased by 33% (HR 1.33, 95% CI 1.14–1.60; *P* < 0.001). However, when the GA/HbA1c ratio was less than 2.21, no significant correlation was detected (HR 0.52, 95% CI 0.26–1.02; *P* = 0.054) (Table 2).

**Table 2:** Threshold effect analysis of GA/HbA1c ratio on all-cause mortality in NAFLD patients

	Adjusted HR (95% CI), *P*-value
All-cause mortality	
Fitting by the standard linear model	1.22 (1.05, 1.42), 0.009
Fitting by the two-piecewise linear model	
Inflection point	2.21
GA/HbA1c ratio < 2.21	0.52 (0.26, 1.02), 0.054
GA/HbA1c ratio ≥ 2.21	1.33 (1.14, 1.60), <0.001
* P* for Log-likelihood ratio	0.015

Analysis was adjusted for age, sex, race, education level, marital status, poverty income ratio, hypertension, diabetes, cardiovascular disease, cancer, smoker, physical activity, estimated glomerular filtration rate, body mass index and nafld fibrosis score

*Abbreviation: HR* Hazard ratio, *CI *Confidence interval

### Other analyses

**Fig. 4:**
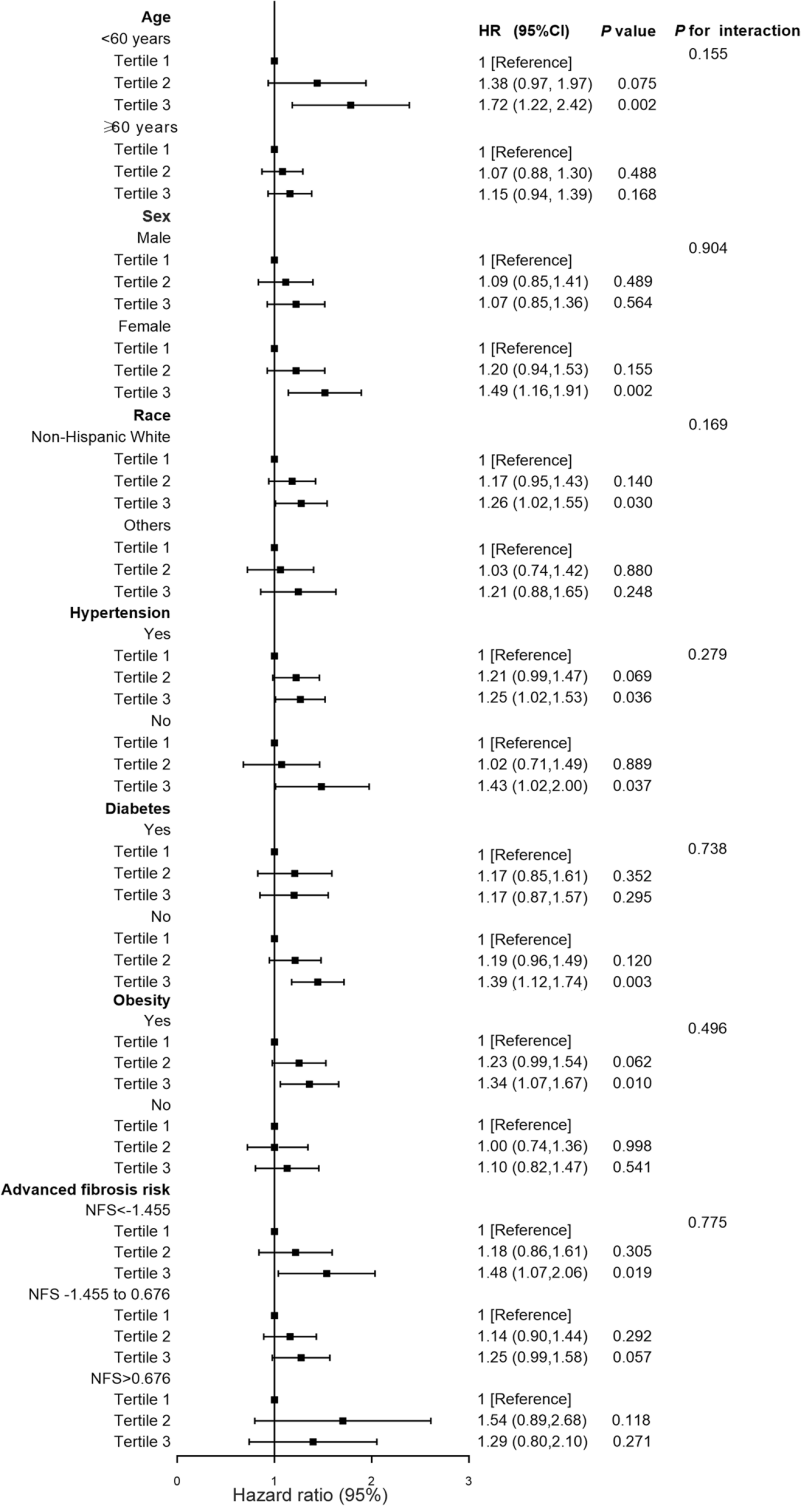
Forest plots of stratified analyses of GA/HbA1c ratio and all-cause mortality. Analysis was adjusted for age, sex, race, education level, marital status, poverty income ratio, hypertension, diabetes, cardiovascular disease, cancer, smoker, physical activity, estimated glomerular filtration rate, body mass index and nafld fibrosis score (Model 3) when they were not the strata variables

To evaluate the consistency of the results and identify potentially different population settings, participants were separated by age, sex, race, hypertension status, diabetes status, obesity status and NFS in a stratified analysis (Fig. 4). A multiplicative interaction term was added to the multivariate model, and the resulting *P* values for the interactions were all > 0.05. The findings indicated that the correlation was not significantly influenced by age, sex, race, hypertension status, diabetes status, obesity status or NFS. According to the sensitivity analyses, the association between GA/HbA1c and all-cause mortality remained almost unchanged, even after excluding the individuals who died within the first two years of follow-up (HR 1.26, 95% CI 1.05–1.51; *P* = 0.012) (Table 3). Moreover, the GA/HbA1c ratio was positively associated with all-cause mortality even after excluding subjects lacking PIR data (HR 1.22, 95% CI 1.02–1.47; *P* = 0.034) (Table 4).

**Table 3:** Association of GA/HbA1c ratio with all-cause mortality after exclusion of participants who died within first 2 years of follow-up

GA/HbA1c ratio	Total	Events	Model 1	Model 2	Model 3
			HR (95%CI), *P*	HR (95%CI), *P*	HR (95%CI), *P*
**All-cause mortality**					
GA/HbA1c ratio (continuous)	5,188	1,364	1.27(1.05,1.53), 0.015	1.25(1.05,1.48), 0.010	1.22(1.03,1.44), 0.022
GA/HbA1c (categorical)					
Tertile 1	1,654	353	1 [Reference]	1 [Reference]	1 [Reference]
Tertile 2	1,797	427	1.14(0.95,1.36), 0.175	1.10(0.92,1.31), 0.315	1.15(0.96,1.38), 0.123
Tertile 3	1,737	584	1.57(1.32,1.87), < 0.001	1.35(1.14,1.60), < 0.001	1.26(1.05,1.51), 0.012
*P* for trend			< 0.001	< 0.001	0.002

Model 1: unadjusted

Model 2: adjusted for age, sex and race

Model 3: adjusted for model 2 plus education level, marital status, poverty income ratio, hypertension, diabetes, cardiovascular disease, cancer, smoker, physical activity, estimated glomerular filtration rate, body mass index and nafld fibrosis score

*Abbreviation: HR *Hazard ratio, *CI *Confidence interval

**Table 4:** Association of GA/HbA1c ratio with all-cause mortality after exclusion of participants with poverty income ratio deficiency

GA/HbA1c ratio	Total	Events	Model 1	Model 2	Model 3
			HR (95%CI), *P*	HR (95%CI), *P*	HR (95%CI), *P*
**All-cause mortality**					
GA/HbA1c ratio(continuous)	4,883	1,330	1.27 (1.05,1.54), 0.015	1.26 (1.06,1.49), 0.009	1.24 (1.05,1.46), 0.011
GA/HbA1c (categorical)					
Tertile 1	1,549	345	1 [Reference]	1 [Reference]	1 [Reference]
Tertile 2	1,692	415	1.16 (0.96,1.39), 0.127	1.12 (0.94,1.35), 0.213	1.15 (0.96,1.38), 0.184
Tertile 3	1,642	570	1.55 (1.30,1.85), < 0.001	1.33 (1.12,1.58), 0.001	1.22 (1.02,1.47), 0.034
*P* for trend			< 0.001	< 0.001	0.004

Model 1: unadjusted

Model 2: adjusted for age, sex and race

Model 3: adjusted for model 2 plus education level, marital status, poverty income ratio, hypertension, diabetes, cardiovascular disease, cancer, smoker, physical activity, estimated glomerular filtration rate, body mass index and nafld fibrosis score

*Abbreviation: HR *Hazard ratio, *CI *Confidence interval

## Discussion

In this study, after including the GA/HbA1c ratio and NAFLD incidence in three cycles of NHANES 1999–2004, we found that the GA/HbA1c ratio was associated with all-cause mortality in NAFLD patients. This study demonstrated an L-shaped curve for the GA/HbA1c ratio and all-cause mortality in subjects with NAFLD, with a cutoff value of 2.21. In individuals with NAFLD below the cutoff, the GA/HbA1c ratio and all-cause death were not correlated. However, a high GA/HbA1c ratio was strongly linked to overall mortality above the cutoff point.

We do not yet fully understand the biological mechanisms that link the GA/HbA1c ratio to mortality. The following mechanisms may be involved. The ratio of GA to HbA1c reflects fluctuations in blood glucose levels. According to the available data, both diabetic and nondiabetic patients may be affected by changes in blood sugar that can result in acute hypoglycemia or large fluctuations in blood glucose after a meal. These changes may induce oxidative stress and hasten the development of atherosclerosis [30, 31]. In addition, glycated albumin is an intermediate of advanced glycosylation end products (AGEs). The pathogenic processes of oxidation and hyaluronic stress are promoted by AGEs, which can also have a negative impact on the body by activating the AGE receptor (RAGE), interfering with cellular signaling, aggregating linked molecules, and interrupting cellular signal conduction [32–34].

In our study, we found an increased risk of mortality in NAFLD patients with a higher GA/HbA1c ratio. However, a previous study showed that in individuals with T2DM, the GA/HbA1c ratio was lower in NAFLD patients than in individuals without NAFLD and was negatively correlated with NAFLD stage [15]. In addition, among 7,117 patients with T2DM, after adjusting for gender, age, and duration of diabetes, there was a significant downward trend in the prevalence of MAFLD based on the quartiles of GA/HbA1C ratio (the Q1, Q2, Q3 and Q4 were 56.3%, 47.4%, 37.8%, and 35.6%, respectively, *P*_trend_ < 0.001), indicating a significant negative correlation [35]. This may be related to the following reasons. First, the previous study focused on T2DM populations, the population we studied was a population of individuals with NAFLD; therefore, there may be differences. Second, the previous study showed a negative correlation between the GA/HbA1c ratio and the progression of hepatic fibrosis, and an elevated GA/HbA1c ratio seems to reduce liver disease-specific mortality; however, since the NHANES restricts the application of liver disease-specific mortality and since the present study focused on all-cause mortality, the GA/HbA1c ratio may affect prognosis in other ways. Our study failed to find any indication of a link between the GA/HbA1c ratio and cardiovascular mortality (likely due to a lack of statistical significance); hence, additional research with larger sample sizes and groups with a high CVD risk are needed. As in previous studies, in the present study, the GA/HbA1c ratio was negatively correlated with BMI [16, 36]. The negative correlation between the GA/HbA1c ratio and BMI may be explained by the following mechanisms. In nondiabetic populations, obesity-related inflammation accelerates protein metabolism, and GA is negatively regulated by BMI [37]. In patients with T2DM, BMI may regulate insulin secretion and thus affect glycemic excursions and/or postprandial hyperglycemia [38].

Our study has several strengths. Due to the use of the national and multistage complex sampling strategy of the NHANES, a strong population representation was guaranteed. In addition, we adjusted for multiple covariates and performed stratified and sensitivity analyses to ensure the stability of the results.

This study has several shortcomings, as follows. First, as the NHANES database contains data from a cross-sectional study, we could determine only the L-shaped relationship between the GA/HbA1c ratio and all-cause mortality in NAFLD participants but not whether there was a causal relationship between the two variables. Second, Liver biopsy is considered the benchmark method for diagnosing liver steatosis. However, since liver biopsy is a test involving invasive procedures, it is not easily accepted for large-scale censuses. Finally, although we adjusted for possible influences as much as possible, it was not possible to take all of them into consideration.

## Conclusion

After adjustment for multivariable factors, the GA/HbA1c ratio exhibited an L-shaped association with all-cause mortality in U.S. patients with NAFLD, with a threshold value of 2.21. When the GA/HbA1c ratio surpassed the threshold, it was notably and positively linked to greater all-cause mortality in patients with NAFLD as the GA/HbA1c ratio increased. This study highlights the significance of the GA/HbA1c ratio in predicting all-cause mortality among NAFLD patients.

## Data Availability

The survey data are publicly available on the internet for data users and researchers throughout the world (www.cdc.gov/nchs/nhanes). Ethical address as the follow: https://www.cdc.gov/nchs/nhanes/irba98.htm.

## Abbreviations

AGEs: Advanced glycosylation end products
ALT: Alanine aminotransferase
AST: Aspartate aminotransferase
BMI: Body mass index
CI: Confidence interval
CVD: Cardiovascular disease
eGFR: Estimated Glomerular filtration rate
FPG: Fasting plasma glucose
GA: Glycated albumin
HbA1c: Hemoglobin A1c
HR: Hazard ratio
HSI: Hepatic steatosis index
NAFLD: Nonalcoholic fatty liver disease
NDI: National death index
NFS: NAFLD fibrosis score
NHANES: National health and nutrition examination survey
PIR: Poverty income ratio
SE: Standard error
T2DM: Type 2 diabetes mellitus
